# Factors associated with the cognitive performance of healthy older adults and persons living with Alzheimer’s disease from a middle‐income country in Latin America

**DOI:** 10.1002/alz.092860

**Published:** 2025-01-09

**Authors:** Leticia Fernanda Palma, Karen Letícia Pulgatti, Pedro Henrique Moreira Victoriano, Laís Rebecchi, Danilo Barroso de Sousa, Marina Mantellatto Grigoli, Cecilia Patricia Popolin, Vanessa Alexandre da Silva, Sabrina Dorta de Oliveira, Patricia Regina Manzine, Márcia Regina Cominetti, Lucas Nogueira de Carvalho Pelegrini

**Affiliations:** ^1^ Federal University of São Carlos, São Carlos, SP Brazil; ^2^ Federal University of São Carlos, São Carlos Brazil; ^3^ Federal University of Sao Carlos, São Carlos Brazil; ^4^ Federal University of Sao Carlos, Sao Carlos, SP Brazil

## Abstract

**Background:**

Low‐ and middle‐income countries (LMIC) share characteristics, such as low education, that increase the risk of dementia among their population. Critically, around 70% of the cases of dementia in LMICs are underdiagnosed. Understanding factors contributing to older adults’ cognitive performance may support healthcare professionals from these countries in their decision‐making process when screening for cognitive decline in their communities. This study aims to analyze factors associated with the cognitive performance of older adults from a Latin American country.

**Method:**

This is a cross‐sectional and analytical study whose participants (n = 92) were healthy older adults (n = 74) and persons living with Alzheimer’s disease (PLwAD) (n = 18) who lived in a middle‐income Latin American country (Brazil). For assessment, the following instruments were used: a sociodemographic questionnaire, the Addenbrooke’s Cognitive Examination‐Revised (ACE‐R), the Mini‐Mental State Examination (MMSE), Geriatric Depressive Scale (GDS‐15), and the Pfeffer Functional Activity Questionnaire (FAQ). Descriptive, linear regression and mediation analyses were performed on SPSS (version 29.0) and PROCESS for SPSS (version 4.1). The significance level was set at p<0.05.

**Result:**

PLwAD were older (t = ‐5.27; p<0.001; 95%CI [‐13.12 to ‐5.75]), had less years of formal education (t = 4.16; p<0.001; 95%CI [2.94 to 8.60]), worse cognitive performance (ACE‐R‐ t = 8.41; p<0.001; 95%CI [31.84 to 52.78] / MMSE‐ t = 9.32; p<0.001; 95%CI [8.96 to 13.82]), and worse functionality (FAQ‐ t = ‐14.31; p<0.001; 95%CI [‐20.25 to ‐15.31]). GDS‐15 scores did not differ between groups (t = ‐0.81; p = 0.423; 95%CI [‐2.27 to 0.93]). Age (B = ‐0.575; t = ‐2.96; p = 0.004), years of education (B = ‐1.073; t = 5.44; p<0.001), and functionality (B = ‐1.593; t = ‐9.99; p<0.001), were significantly associated with cognitive performance, as per ACE‐R scores (R^2^ = 0.799; F = 102.27; p<0.001). Finally, mediation analysis suggested that years of education mediate the effect of FAQ on ACE‐R scores (total effect = ‐2.15; p<0.001 / direct effect = ‐1.78; p<0.001 / indirect effect = ‐0.3558; 95%BCaCI [‐0.61 to ‐0.18]).

**Conclusion:**

These findings align with existing literature, yet they present a novel perspective within this population. Results demonstrate that age, education, and functionality are associated with cognitive performance, emphasizing their relevance in the screening/assessment. Our data also suggests that years of education mediate the relationship between functionality and cognitive performance.

